# Pressão Arterial de Crianças: Associação a Indicadores Antropométricos, Composição Corporal, Aptidão Cardiorrespiratória e Atividade Física

**DOI:** 10.36660/abc.20190520

**Published:** 2021-05-06

**Authors:** Gisele Pinheiro, Júlio Mello, Adroaldo Gaya, Anelise Reis Gaya

**Affiliations:** 1 Universidade Federal do Rio Grande do Sul Porto AlegreRS Brasil Universidade Federal do Rio Grande do Sul, Porto Alegre, RS – Brasil.; 2 Faculdade SOGIPA de Educação Física Porto AlegreRS Brasil Faculdade SOGIPA de Educação Física, Porto Alegre, RS - Brasil.

**Keywords:** Criança, Pressão Arterial, Antropometria, Exercício, Composição Corporal, Aptidão Física, Atividade Motora

## Abstract

**Fundamento:**

Evidências apontam variáveis antropométricas e de condicionamento físico como fatores associados à pressão arterial infantil. Analisá-los em apenas um contexto é um meio relevante de identificar o peso que cada um deles pode apresentar no desenvolvimento da hipertensão arterial.

**Objetivo:**

Identificar as possíveis associações de medidas antropométricas, da composição corporal, da atividade física moderada-vigorosa (AFMV) e da aptidão cardiorrespiratória (ApC) com a pressão arterial em crianças.

**Métodos:**

Estudo correlacional com abordagem quantitativa. Duzentos e quinze (215) estudantes com idades de 6 a 12 anos de uma escola pública de Porto Alegre, selecionados por critério de conveniência. A pressão arterial foi aferida através de um esfigmomanômetro digital. Para o tratamento dos dados, os valores de pressão arterial sistólica e diastólica foram padronizados (escore Z) e somados. As variáveis testadas como preditoras foram: AFMV; Percentual de gordura (%G); Índice de massa corporal (IMC); Razão cintura/estatura (RCE); Maturação somática; ApC. Após a verificação dos parâmetros de normalidade, as associações brutas e ajustadas (para sexo, idade e maturação somática) foram testadas através de equações de regressão linear. Para as análises, foi considerado p < 0,05.

**Resultados:**

Três diferentes modelos indicaram os melhores conjuntos de fatores associados à pressão arterial padronizada: O Modelo 1 (R^2^ = 0,21) se constituiu das variáveis RCE (β = 9,702) e AFMV (β = – 0,021); O Modelo 2 (R^2^ = 0,19) foi composto pelas variáveis IMC (β = 0,156) e AFMV (β = – 0,021); O Modelo 3 (R^2^ = 0,18) incluiu as variáveis %G (β = 0,063) e ApC (β = – 0,004).

**Conclusões:**

A pressão arterial de crianças é predita pelas variáveis corporais %G, IMC e RCE. Além disso, está associada negativamente à AFMV e a ApC.

## Introdução

A pressão arterial é um importante indicador da saúde cardiovascular e metabólica. Crianças com níveis elevados de pressão arterial têm alta probabilidade de se tornarem adultos hipertensos. Portanto, o diagnóstico e tratamento precoce podem evitar eventos cardiovasculares adversos a longo prazo.^1^ Apesar de a hipertensão arterial ser mais frequente na vida adulta, evidências epidemiológicas sugerem que a sua gênese possa estar localizada na infância.^2^ Todavia, cabe ressaltar que pesquisas recentes demonstram índices consideráveis da prevalência de elevados valores de pressão arterial.^3^

Para compreendermos a hipertensão na infância e adolescência, é de suma importância considerarmos variáveis como: idade, estatura, sexo, sobrepeso/obesidade, níveis de atividade física e níveis de aptidão física.^4^^–^^6^ O estudo de Freedman et al.,^7^ sugeriu que crianças com sobrepeso ou obesidade a mais chances de apresentarem níveis de pressão arterial elevados. Pesquisas mostram uma associação negativa entre o nível de atividade física e a pressão arterial,^3^ assim como outros estudos^8^^,^^9^ têm demonstrado que crianças e adolescentes com baixos níveis de aptidão cardiorrespiratória acrescido a um excesso de peso possuem mais chances de apresentar fatores de risco para doenças cardiovasculares.

Evidências sobre todos os fatores avaliados em um mesmo estudo agregam informações relevantes sobre a magnitude da influência que cada indivíduo pode apresentar para o desenvolvimento da hipertensão arterial. Desse modo, o presente estudo tem como objetivo verificar as possíveis associações do conjunto de medidas antropométricas, de composição corporal, de atividade física moderada-vigorosa e de aptidão cardiorrespiratória com a variabilidade da pressão arterial em crianças.

## Métodos

### Caracterização do Estudo

Trata-se de um estudo de corte transversal com método correlacional e abordagem quantitativa.^10^

### Sujeitos da Pesquisa

A população-alvo^11^ são estudantes de primeiro a quinto ano do ensino fundamental, com idade entre 6 a 12 anos e matriculados em escolas públicas. A população disponível^11^ foi composta por aproximadamente 400 crianças estudantes do 1º ao 5º ano do ensino fundamental de uma escola pública estadual de Porto Alegre. A população disponível foi selecionada por conveniência, justificada pelo fato desta escola atender a disciplina de estágio supervisionado do curso de Educação Física na mesma universidade frequentada pelos pesquisadores e, como tal, permitir pleno acesso dos mesmos à comunidade escolar.

Para compor a amostra foram convidadas todas as crianças matriculadas nos anos iniciais do ensino fundamental. Foram avaliadas 215 crianças de 6 a 12 anos de idade. Após a coleta de dados, para identificar a dimensão amostral que mais equilibra a probabilidade entre os erros de tipo I e II, respectivamente, e para justificar a testagem estatística em uma amostra não aleatória, foi utilizado o programa G-Power, versão 3.1.

Foi feito o cálculo para testes da família F, mais especificamente o de regressão linear múltipla. O alpha utilizado foi 0,05, o tamanho de efeito de foi 0,15 (médio), o poder do teste foi de 0,95 e o número de preditores foi 8 (oito) (considerando o modelo com o maior número de variáveis possível neste estudo: razão cintura-estatura (RCE), índice de massa corporal (IMC), percentual de gordura (%G), atividade física moderada-vigorosa, aptidão cardiorrespiratória, sexo, idade e maturação somática). A partir destes critérios foi estipulada uma dimensão amostral mínima de 160 sujeitos.

A coleta de dados foi realizada no período entre março e abril de 2017. A escola foi convidada a participar do estudo e consentiu através de uma Carta de Anuência. Em seguida, foi convocada e realizada uma reunião com os pais para detalhar o estudo. Por fim, estes receberam e assinaram um Termo de Consentimento do Livre e Esclarecido. Os alunos, por sua vez, assinaram um Termo de Assentimento. Ambos os termos explicavam o estudo. A pesquisa foi aprovada pelo Comitê de Ética em Pesquisa com Humanos da Universidade Federal do Rio Grande do Sul (UFRGS) sob o parecer nº 2.571.198.

### Procedimento de Coleta de Dados

As variáveis antropométricas (estatura, massa e perímetro da cintura) foram avaliadas seguindo as recomendações do Manual de Medidas e Testes do Proesp-Br.^12^ A massa corporal foi avaliada com os escolares descalços, através de uma balança digital portátil (*Tech Line*) e cuja precisão era de 100 g. Os valores foram aferidos em Kg, com o uso de uma casa decimal.

Para a medição da estatura foi utilizada uma fita métrica com precisão de 2 mm, presa verticalmente à parede a 1 m do solo e estendida de baixo para cima. Os resultados foram aferidos em cm, com o uso de uma casa decimal e o auxílio de um esquadro para a leitura. A medida de perímetro da cintura foi aferida através de uma fita métrica flexível posicionada no ponto médio entre a borda inferior da última costela e a crista ilíaca, geralmente próxima da cicatriz umbilical. As demais variáveis antropométricas seguiram as recomendações de Mirwald et al.,^13^ A altura do indivíduo quando sentado foi aferida com um banco padrão para todas as crianças e uma fita métrica com precisão de 2 mm fixada à parede. O ponto-zero da fita métrica foi fixado no acento do banco.

Para o cálculo do IMC foram utilizadas as variáveis estatura e massa corporal, para o cálculo da RCE foram utilizadas as variáveis estatura e perímetro da cintura.^12^ Para o cálculo da maturação somática foram utilizadas as variáveis sexo, estatura, massa corporal, estatura sentada, comprimento da perna (diferença entre estatura total e estatura sentado) e idade.^13^

A aferição da pressão arterial foi realizada durante o período de aulas da escola, através do uso de um esfigmomanômetro digital com braçadeiras adequadas da marca Omron. Todas as verificações de medidas foram feitas entre o primeiro e o segundo período escolar matutino (entre oito e nove horas), período em que nem todas as crianças estavam em jejum. Foi solicitado aos alunos que se mantivessem sentados por cinco minutos para que se pudesse obter um valor mais próximo do repouso. Foram feitas três aferições no braço direito e, em seguida, computado o valor médio. Os valores de pressão arterial sistólica e diastólica foram padronizados (através do uso de escore Z). O escore Z da pressão arterial sistólica foi somado ao escore Z da pressão arterial diastólica, o que resultou em uma nova variável da pressão arterial padronizada.

Para mensurar o percentual de gordura, foi utilizado o exame de imagem por absorciometria de duplo feixe de raios X (DXA) da marca GE Healthcare, modelo Lunar Prodigy e realizado por um pesquisador treinado. As crianças foram instruídas a vestirem roupas sem zíperes, fechos e fivelas, a removerem qualquer peça de metal, a se posicionarem deitadas em decúbito dorsal e permanecerem paradas até o braço do aparelho passar sobre o corpo no trajeto entre cabeça e os pés. Os valores, em percentuais, foram calculados automaticamente pelo *software* do equipamento.

Para mensurar a atividade física foi utilizado um acelerômetro Actigraph (wActiSleep-BT Monitor), que foi posicionado na cintura dos escolares através de um cinto elástico na linha axilar média do lado direito. As crianças foram incentivadas a usá-lo durante sete dias consecutivos. Para fins de análise, foram considerados cinco dias (incluso um dia do final de semana) e pelo menos 10 horas por dia de tempo de uso. O equipamento foi mantido durante todo o dia e retirado apenas para o banho ou quaisquer outras atividades aquáticas. Após o último dia de uso do acelerômetro, o aparelho foi retirado pela equipe avaliadora. Posteriormente, através do *software* Actilife (ActiGraph^®^, version 5.6, EUA) foi verificado se os dados estavam completos.

Os dados foram coletados em uma taxa de amostragem de 30 Hz, baixados em períodos de um segundo e agregados em períodos de 15 segundos. Na apuração de *counts* para os pontos de corte em acelerômetros, foi utilizada a proposta de Evenson et al.^14^ para períodos de 15 segundos: ≤ 25 counts/15 segundos para o tempo sedentário; ≥ 574 counts/15 segundos para atividade física moderada e ≥ 1.003 counts/15 segundos para atividade física vigorosa.

Para medir a aptidão cardiorrespiratória foi feito o teste de corrida/caminhada de seis minutos, conforme as recomendações do Manual de Medidas e Testes do Proesp-Br.^12^ Para a sua realização, foi utilizada uma quadra com perímetro de 56 metros, demarcada com cones e sinalizadas com giz a cada dois metros para aferir a distância percorrida por cada criança ao final do teste. Foi utilizado também um cronômetro e um apito para iniciar e finalizar a atividade. As crianças foram instruídas a percorrer (correr ou caminhar) o maior número de voltas, ou seja, a maior distância possível. Durante o teste, foi avisada a passagem do tempo aos dois, quatro e cinco minutos, respectivamente, (“Atenção: falta um minuto!”) e ao final da atividade (com o som do apito). As crianças deveriam parar no lugar em que estavam no momento do apito e permanecer neste ponto até que o avaliador anotasse a distância percorrida.

### Procedimentos Estatísticos

Inicialmente, todas as variáveis foram submetidas ao Teste de Normalidade de Kolmogorov-Smirnov e demonstraram distribuição normal. Para a análise descritiva, foram utilizados valores médios e desvios padrão, mínimo e máximo para as variáveis contínuas, além de valores de frequência absoluta e relativa para as variáveis categóricas. A diferença entre os sexos foi testada em todas as variáveis com o Teste *t* de Student para amostras independentes. De início, as associações foram testadas com o Teste de Correlação de Pearson. A multicolineariedade foi testada previamente e foi identificada uma alta relação entre a atividade física de moderada-vigorosa e a aptidão cardiorrespiratória. Estes procedimentos foram realizados para atender os pressupostos da regressão linear múltipla. A análise seguinte se propôs a estimar a variância da pressão arterial padronizada a partir das demais variáveis estudadas.

Foram testados diferentes modelos de regressão linear múltipla a partir do método Stepwise. Aqueles que apresentaram maiores valores de R^2^ ajustado foram considerados, desde que mantivessem a lógica teórica. Todas as associações foram ajustadas quanto ao sexo, a idade e a maturação somática, no intuito de retirar o efeito de possíveis confundidores. Para as análises, foi considerado *a priori* um *alpha* de 0,05 e foram realizadas com o *software* SPSS para Windows, versão 20.0.

## Resultados

A amostra revelou que, no total, 53,5% são meninos e 46,5% são meninas. Na Tabela 1, são apresentadas as características dos sujeitos da pesquisa, relacionadas com as variáveis estudadas. Apenas as variáveis “percentual de gordura”, “atividade física moderada-vigorosa” e “aptidão cardiorrespiratória” apresentaram diferenças entre os sexos (p < 0,05). Importante destacar o baixo desempenho no teste de corrida/caminhada de seis minutos em ambos os sexos.

**Tabela 1:** Descrição das características dos sujeitos da pesquisa (n = 215)

	MENINOS					MENINAS				
	N	X ± DP	Min	Max	N		X ± DP	Min	Max	Valor de p
**Idade (anos)**	115	8,25 ±1,54	6	12	100		8,51 ± 1,44	6	11	0,211
**PAS (mmHg)**	115	103,04 ± 11,35	83	143	100		103,81 ± 11,75	77	134	0,628
**PAD (mmHg)**	115	60,78 ± 9,13	41	81	100		60,67 ± 8,59	42	82	0,926
**Estatura (cm)**	111	134,27 ± 10,09	111	161	98		134,47 ± 10,84	108	154	0,890
**Peso (kg)**	111	32,98 ± 9,58	18	61	98		33,82 ± 11,23	15	67	0,558
**PC (cm)**	111	63,41 ± 9,09	48	86	98		61,75 ± 10	35	90	0,209
**% G**	55	31,86 ± 9,09	15,6	51,2	57		35,24 ± 7,55	17,2	49,6	**0,035**
**IMC (kg/m²)**	111	18 ± 3,47	12,4	29,5	98		18,27 ± 3,98	12,6	29,78	0,600
**RCE**	111	0,47 ± 0,05	0,37	0,64	98		0,45 ± 0,05	0,27	0,60	0,800
**AFMV (min)**	57	70,26 ± 29,57	23,62	147,16	60		55,33 ± 18,75	23,64	110,24	**0,002**
**ApC (m)**	101	800,43 ± 142,99	438	1158	91		749,25 ± 104,64	504	952	**0,005**

Fonte: os autores. N: número de sujeitos; X: média; DP: desvio padrão; PAS: pressão arterial sistólica; PAD: pressão arterial diastólica; PC: perímetro da cintura; %G: percentual de gordura; IMC: índice de massa corporal; RCE: razão cintura/estatura; AFMV: atividade física moderada-vigorosa; ApC: aptidão cardiorrespiratória.

A análise de correlação está apresentada na Tabela 2. A pressão arterial padronizada está associada de forma negativa e positiva com algumas variáveis nos meninos, todavia com índices de correlação (r) razoavelmente baixos. Já nas meninas, todos os valores se encontram abaixo de 0,3, o que indica que na análise bivariada, as associações são fracas.

**Tabela 2:** Valores da correlação entre a pressão arterial padronizada e a composição corporal, a atividade física moderada-vigorosa e a aptidão cardiorrespiratória (n = 215)

	zPA	
	MENINOS (n:115)	MENINAS (n:100)
	r	r
**RCE**	0,368	0,252
**IMC**	0,454	0,342
**%G**	0,490	0,288
**AFMV**	-0,349	-0,253
**ApC**	-0,216	-0,227

zPA: pressão arterial padronizada; r: coeficiente da correlação; RCE: razão cintura/estatura; IMC: índice de massa corporal; %G: percentual de gordura; AFMV: atividade física moderada-vigorosa; ApC: aptidão cardiorrespiratória.

Após a verificação das correlações, foram feitas as análises de associação ajustadas. Os resultados mostraram que, considerando o sexo, a idade e a maturação somática constantes, as variáveis “razão cintura/estatura”, “índice de massa corporal” e “percentual de gordura” indicam uma associação positiva com a pressão arterial padronizada. Em contrapartida, a atividade física moderada-vigorosa e aptidão cardiorrespiratória indicaram uma relação negativa.

As variáveis preditoras foram testadas em diversos modelos. Entretanto, três diferentes modelos indicaram os conjuntos de fatores que melhor explicaram a variância da pressão arterial padronizada (Tabela 3). Embora os três modelos sejam compostos por variáveis diferentes, explicaram entre 18% e 21% da variância da pressão arterial padronizada. Outro importante fator é que as duas medidas de estado nutricional (índice de massa corporal e razão cintura/estatura) foram os indicadores que apresentaram maiores magnitudes de associação, considerando os ajustes já descritos.

**Tabela 3:** Regressão linear múltipla para a estimativa da variabilidade da pressão arterial padronizada a partir de 3 modelos (n = 215)

zPA			
Modelo 1 (R^2^ ajustado: 0,210)			
	β	p	IC 95%
**RCE**	7,170	0,022	1,033 – 13,308
**AFMV**	-0,021	0,004	-0,035 – -0,007
**Sexo**	-0,998	0,136	-2,315 – 0,320
**Idade**	-0,302	0,212	-0,778 – 0,174
**Maturação**	0,456	0,079	-0,054 – 0,965
**Modelo 2 (R^2^ ajustado: 0,192)**			
**IMC**	0,113	0,090	-0,018 – 0,245
**AFMV**	-0,023	0,002	-0,037 – -0,008
**Sexo**	-0,697	0,408	-2,360 – 0,967
**Idade**	-0,254	0,364	-0,808 – 0,299
**Maturação**	0,260	0,471	-0,453 – 0,973
**Modelo 3 (R^2^ ajustado: 0,183)**			
**%G**	0,054	0,043	0,002 – 0,107
**ApC**	-0,003	0,037	-0,006 – 0,001
**Sexo**	-0,737	0,307	-2,163 – 0,689
**Idade**	0,090	0,765	-0,508 – 0,688
**Maturação**	0,238	0,466	-0,408 – 0,884

zPA: pressão arterial padronizada; R^2^ ajustado: coeficiente de determinação ajustado para as variáveis do modelo; β: associação ajustada para sexo, idade e maturação somática; p: nível de significância; RCE: razão cintura/estatura; AFMV: atividade física moderada-vigorosa; IMC: índice de massa corporal; %G: percentual de gordura; ApC: aptidão cardiorrespiratória.

## Discussão

Os principais resultados do presente estudo indicaram que a atividade física moderada-vigorosa, a aptidão cardiorrespiratória, as variáveis antropométricas, a idade, o sexo e a maturação somática são importantes preditores da variabilidade da pressão arterial padronizada nas crianças. Em nossos resultados, observamos que a média da pressão arterial sistólica foi de 103,0 mmHg em meninos e de103,8 mmHg em meninas. A pressão arterial diastólica foi de 60,7 mmHg em meninos e de 60,6 mmHg em meninas.

No estudo de Gaya et a.,^15^ com 416 meninos portugueses entre 8 e 15 anos, a média da pressão arterial sistólica foi de 117,2 mmHg e da pressão arterial diastólica foi de 61,1 mmHg. Já no estudo de Monteiro et al.,16. com 51 escolares brasileiros, a média da pressão arterial sistólica foi de 111,6 mmHg em meninos e 107,6 em meninas, e diastólica de 66,8 mmHg em meninos e 66,5 em meninas com idades entre 13 e 16 anos. Uma possível explicação para os valores de pressão arterial sistólica serem maiores nos estudos supracitados em comparação com os aferidos pode ser a idade cronológica dos sujeitos serem maiores. Isto influencia diretamente nos níveis pressóricos, já que as crianças mais velhas tendem a ter uma maior estatura, o que naturalmente eleva os níveis pressóricos.^2^

No que diz respeito à aptidão cardiorrespiratória, percebemos que a média de metros percorridos no teste de seis minutos é baixa para meninos e meninas. No estudo de Mello et al.,^17^ ainda que tenha sido com crianças e adolescentes entre 10 a 17 anos, a prevalência de baixa aptidão cardiorrespiratória foi de 74,1% entre os jovens sendo superior nas meninas. Mesmo utilizando os critérios do Proesp-BR, iguais aos deste estudo, os resultados encontrados por Mello et al.,^17^ foram mais preocupantes. Todavia, nos estudos de Coledam et al.,^18^ e Minatto et al.,^19^ os autores observaram resultados semelhantes ao presente estudo, mostrando que em torno de 50% das crianças apresentavam baixos níveis de aptidão cardiorrespiratória.

Já em relação à atividade física moderada-vigorosa, no presente estudo observamos uma média de 70,2 min/sem nos meninos e de 55,3 min/sem em meninas. Matsudo et al.,^20^ observou em crianças brasileiras de 9 a 11 anos de idade uma média de atividade física moderada-vigorosa de 59,5 min/dia, sendo que as crianças acumularam mais atividade deste tipo nos dias úteis que nos finais de semana. Ademais, 55,9% não atingiram a recomendação diária estipulada.

Percebemos que as meninas tendem a possuir valores menores, tanto de aptidão cardiorrespiratória quanto de atividade física moderada-vigorosa. Esse dado pode ser explicado por um conjunto de fatores relacionados com a cultura de incentivo para a prática de atividades físicas vigorosas, variáveis antropométricas, étnicas e fisiológicas.^21^ Treuth et al.^22^ apontou em um estudo realizado com meninas que a atividade mais realizada durante a semana e na maior parte dos dias de finais de semana são de caráter sedentário (55,4% do tempo). Dentro do tempo em atividade, eram praticadas em sua maioria atividades com baixa intensidade (41,7% atividades leves) e pouco tempo era dispensado em atividades moderadas (2,2%) e vigorosas (0,7%).

No tocante às associações, nossos resultados iniciais demonstraram uma correlação entre a pressão arterial padronizada e todos os indicadores de sobrepeso e obesidade, bem como a atividade física moderada-vigorosa e aptidão cardiorrespiratória. Todos os resultados apresentaram correlações de magnitude moderada ou baixa (r < 0,4). Essa baixa magnitude pode ser explicada principalmente pelos diversos fatores que influenciam na pressão arterial, como a estatura, o sexo e a idade,^23^ que não fizeram parte desta análise inicial. Portanto, a partir dessas primeiros estudos se fez clara a necessidade de análises ajustadas.

Nesta perspectiva, foram analisados modelos que pudessem explicar parte da variância da pressão arterial padronizada, mediante o ajuste de associações para as variáveis “maturação somática”, “idade” e “sexo”. Dessa forma, percebemos de forma mais clara o quanto as variáveis: “razão cintura/estatura”, “índice de massa corporal”, “percentual de gordura”, “atividade física moderada-vigorosa” e “aptidão cardiorrespiratória” podem, de fato, influenciar na pressão arterial padronizada dos escolares, tanto de forma individual (através dos valores de beta) quanto de forma conjunta (através dos valores de R^2^ ajustados).

Partindo deste ponto, foram testadas as variáveis preditoras, o que resultou em três modelos que melhor explicam a variância da pressão arterial padronizada. Os dois primeiros (modelo 1: razão cintura/estatura, atividade física moderada-vigorosa, sexo, idade e maturação; e modelo 2: índice de massa corporal, atividade física moderada-vigorosa, sexo, idade e maturação) explicam 21% e 19% da variância, respectivamente. A variável “razão cintura/estatura”, que é um indicador de gordura abdominal, é a que mais influência no grau de variância da pressão arterial. Esses resultados vão de encontro a estudo de Silva et al.,^24^ que demostrou a associação do excesso de gordura visceral com os índices de pressão arterial e a prevalência de hipertensão arterial. Entretanto, segundo o estudo de Cauduro et al.,^25^ crianças e adolescentes obesos teriam menos chance de apresentar níveis elevados de pressão arterial independentemente do sexo, da idade e do nível socioeconômico, caso cumprissem as recomendações de atividades físicas ideais para a sua idade.

Portanto, os resultados de Cauduro et al.,^25^ demonstram a importância de considerar a atividade física e algum indicador de sobrepeso e obesidade no mesmo modelo de associação, diante da perspectiva de tentarmos entender o conjunto de preditores da pressão arterial em crianças. Nossos resultados ainda demostram que, independentemente do indicador de sobrepeso/obesidade inserido no modelo, as associações se mantêm. Isso se justifica pelo fato de ambas as variáveis estarem associadas de forma independente à pressão arterial.^6^^,^^26^^,^^27^

Já no último modelo apresentado, observamos que a aptidão cardiorrespiratória e o percentual de gordura explicam 18% da variância da pressão arterial padronizada. É importante salientar que, apesar de a magnitude da associação da aptidão cardiorrespiratória ser baixa em relação à pressão arterial padronizada (β: -0,003), aparentemente, percebemos pela unidade de medida dessa variável ser metros que se trata de um resultado importante e que pode influenciar facilmente na pressão arterial de crianças.

Por fim, é importante acrescentar que a aptidão cardiorrespiratória e atividade física moderada-vigorosa não estão no mesmo modelo, visto que ambas se associam teoricamente.^28^ Nos testes de multicolineariedade, a relação encontrada no presente estudo foi de r > 0,7. Ainda é importante salientar que mesmo com três indicadores de sobrepeso/obesidade realizados através de medidas diferentes (crescimento corporal, gordura abdominal e exame de imagem) as associações se mantiveram e os modelos permaneceram em um percentual de explicação da pressão arterial padronizada semelhante. Ademais, também ressaltamos que no ambiente escolar a utilização de medidas antropométricas para estimar o estado nutricional de crianças é uma estratégia eficaz e possui um efeito de análise semelhante à avaliação por imagem. Isso indica que estas variáveis (índice de massa corporal e razão cintura/estatura) podem ser agregadas aos programas de intervenção.

## Conclusão

Percebemos que as variáveis corporais: “percentual de gordura”, “índice de massa corporal” e “razão cintura/estatura” se mostraram influentes à pressão arterial. Além disso, observou-se que a atividade física moderada-vigorosa e a aptidão cardiorrespiratória, que são importantes variáveis relacionadas com o exercício, também influenciam na pressão arterial de crianças. Portanto, podemos concluir que todos os indicadores estudados, quando analisados em conjunto, se associam à pressão arterial de crianças, o que sugere que a prevenção precoce de hipertensão arterial em crianças deve considerar a prática regular de atividade física moderada-vigorosa, os aumentos dos níveis de aptidão cardiorrespiratória e as estratégias a serem adotadas para o controle dos indicadores de sobrepeso e obesidade.
