# Supplementary material for: Pressão Arterial de Crianças: Associação a Indicadores Antropométricos, Composição Corporal, Aptidão Cardiorrespiratória e Atividade Física
Source: Arq Bras Cardiol. 2021 May 6;116(5):950–6. [Article in Portuguese] doi: 10.36660/abc.20190520 (PMC8121480; doi:10.36660/abc.20190520)
Supplement: Translation

# Translation: Blood Pressure in Children: Association with Anthropometric Indicators, Body Composition, Cardiorespiratory Fitness and Physical Activity

**Authors:** Gisele Pinheiro, Júlio Mello, Adroaldo Gaya, Anelise Reis Gaya

**Affiliations:** 1 Porto AlegreRS Brazil Universidade Federal do Rio Grande do Sul, Porto Alegre, RS – Brazil.; 2 Porto AlegreRS Brazil Faculdade SOGIPA de Educação Física, Porto Alegre, RS - Brazil.

**Keywords:** Child, Blood Pressure, Anthropometry, Exercise, Body Composition, Physical Fitness, Motor Activity

## Abstract

**Background:**

Evidence points to anthropometric and fitness variables as associated factors with children's blood pressure. Analysing these factors in a single context is a relevant possibility of identifying the weight that each factor can present for the development of arterial hypertension.

**Objective:**

Identify the possible associations between anthropometric measurements, body composition, moderate-vigorous physical activity (MVPA) and cardiorespiratory fitness (CRF) with blood pressure in children.

**Methods:**

Correlational study with a quantitative approach. Sample: 215 schoolchildren aged 6-12 years selected by convenience criteria of a public school in Porto Alegre, Brazil. Blood pressure was measured with a digital sphygmomanometer. For data treatment, the values of systolic and diastolic blood pressure were standardized (Z score) and added. The variables tested as predictors were: MVPA; body fat percentage (BF%); Body Mass Index (BMI); waist-height ratio (WHTR); maturity-offset and CRF. After checking the normality parameters, the crude and adjusted associations (for sex, age and maturity-offset) were tested with linear regression equations. For the analyses, p <0.05 was considered.

**Results:**

Three different models indicated the best sets of factors associated with standardized blood pressure. Model 1 (R^2^ = 0.21) consisted of the variables WHTR (β = 9.702) and MVPA (β = −0.021). Model 2 (R^2^ = 0.19) was composed of the variables BMI (β = 0.156) and MVPA (β = −0.021). Model 3 (R^2^ = 0.18) included the variables BF% (β = 0.063) and CRF (β = −0.004).

**Conclusion:**

Blood pressure in children is predicted by the body variables BF%, BMI and WHTR, in addition, it is negatively associated with MVPA and CRF.

## Introduction

Blood pressure is an important indicator of cardiovascular and metabolic health. Children with high blood pressure levels are highly likely to become hypertensive adults, then, the early diagnosis and treatment can prevent long-term adverse cardiovascular events.^1^ Although arterial hypertension is more frequent in adulthood, epidemiological evidence suggests that its genesis may be in childhood.^2^ However, it is noteworthy that recent researches have shown considerable prevalence rates of high blood pressure values.^3^

In order to understand hypertension in childhood and adolescence, it is relevant to consider variables such as: age, height, sex, overweight/obesity, physical activity and fitness levels.^4^^–^^6^ The study by Freedman et al.^7^ suggested that overweight or obese children are more likely to have high blood pressure. Other studies have shown a negative association between the level of physical activity and blood pressure.^3^ As well as other studies^8^^,^^9^ have shown that children and adolescents with low cardiorespiratory fitness levels plus overweight/obesity have a greater chance of presenting cardiovascular diseases risk factors.

In this sense, evidence about all factors evaluated together in the same study adds relevant information on the magnitude of the influence that each one can present for the development of arterial hypertension. In this context, the present study aims to identify the possible associations between anthropometric measurements, body composition, moderate-vigorous physical activity and cardiorespiratory fitness with blood pressure in children

## Methods

### Study Design

This is a cross-sectional study with correlational method and quantitative approach.^10^

### Research Subjects

Target population^11^ are students from the first to fifth year of elementary school, aged between 6-12 years enrolled in public schools. The available population^11^ was composed of approximately 400 schoolchildren from the first to fifth year of elementary school at a public school at Porto Alegre, Brazil. The available population was selected for convenience, justified for this school meets the supervised internship subject in the Physical Education graduation of the researchers’ university and, as such, allows full access of researchers to the school community.

To compose the sample, all children enrolled in the early five years of elementary school were invited. 215 children from 6-12 years old were evaluated. After data collection, to identify the sample size that most balances the probability between type I and II errors and to justify the use of statistical tests in a non-random sample, the G-Power version 3.1 program was used. The calculation was performed for F family tests, more specifically multiple linear regression, the alpha used was 0.05, the effect size was 0.15 (medium), the test power was 0.95 and the number of predictors was eight (considering the model with the greatest possible number of variables in this study: waist-to-height ratio (WHTR), Body Mass Index (BMI), body fat percentage (BF%), moderate-vigorous physical activity, cardiorespiratory fitness, sex, age and maturity-offset). From these criteria, a minimum sample size of 160 subjects was stipulated.

Data collection was carried out between March and April 2017. The school was invited to participate in the study and consented through a consent letter. Afterwards, a meeting was held with the parents detailing the study and then, they received and signed a Free and Informed Consent Form and the students signed an Informed Assent Form, both terms explaining the study. The research was approved by the Human Research Ethics Committee of the Universidade Federal do Rio Grande do Sul,, Brazil under number 2.571.198.

### Data Collection Procedures

Anthropometric variables (height, mass and waist circumference) were assessed following the Guidelines for Measurement and Tests of PROESP-Br.^12^ Body mass was assessed with the students barefoot using a portable digital scale (Tech Line) with 100g precision and the value in Kg was recorded using a decimal place. To measure height, a measuring tape with two millimetres precision was used, which was attached to the wall one meter from the ground vertically, extended from the bottom up and noted in centimetres with a decimal point with the aid of a square for reading. Waist circumference was measured using a measuring tape, positioning it at the midpoint between the lower edge of the last rib and the iliac crest, usually close to the umbilical scar. The other anthropometric variables followed the recommendations of Mirwald et al.^13^ The seated height was measured with a standard bench for all children and a measuring tape with two millimetres precision fixed to the wall. The zero point of the measuring tape was fixed to the bench.

Height and body mass were used to calculate BMI, while height and waist circumference were used to calculate WHTR.^12^ For the maturity-offset calculation, the variables sex, height, body mass, sitting height, leg length (the difference between total height and sitting height) and age were used.^13^

Blood pressure was measured during the school's class period using a digital sphygmomanometer with appropriate OMRON cuffs. All measurements were carried out between the first and the second school period in the morning (≈8-9h), so in general, all children were not fasting. Children were invited to remain seated for five minutes to obtain a value closer to rest. Three measurements were performed on the right arm and the average value was computed. The values of systolic and diastolic blood pressure were standardized (using Z score). The Z score of systolic blood pressure was added to the Z score of diastolic blood pressure, creating a new standardized blood pressure variable.

Body fat percentage was assessed by the imaging exam of Dual Energy X-ray Absorptiometry (DXA) of the GE Healthcare model Lunar Prodigy, performed by a trained researcher. Children were instructed to wear clothes without zippers and buckles; to remove any metal piece; to lying in the supine position and; remain stationary until the device arm passes over the body in the head-to-foot direction. The values, in percentage, were calculated automatically by the equipment software.

ActiGraph accelerometers (wActiSleep-BT Monitor) were used to measure physical activity, which was placed on the students’ waist on an elastic belt on the right midline axillary. Children were encouraged to use it for seven consecutive days. For analysis purposes, five days (including one weekend day) were considered, with at least 10 hours/day of usage time. The equipment was maintained throughout the day and removed only for bathing or any water activities. After the last day of use, the device was removed by the evaluation team and subsequently it was verified if data were complete, using the Actilife software (ActiGraph®, version 5.6, USA). The data were collected at a sampling rate of 30Hz, downloaded in periods of one second, and aggregated for periods of 15 seconds. Everson et al.^14^ proposal for 15 seconds periods was used for classifying the accelerometers counts (≤25 counts/15 seconds for sedentary time, ≥574 counts/15 seconds for moderate physical activity and ≥1003 counts/15 seconds for vigorous physical activity).

For the measurement of cardiorespiratory fitness, the six-minute running/walking test was performed according to the Guidelines for Measurement and Tests of PROESP-Br.^12^ To perform this test, a court with a 56-meter perimeter marked with cones and marked with chalk every two meters was used to record the distance covered by each child at the end of the test. A stopwatch and a whistle were also used to start and end the test. Children were instructed to run (run or walk) the largest number of laps, that is, the longest distance possible. During the test, the evaluators informed the passage of the test time in two, four and five minutes (“attention: one minute left”) and at the end of the test (at the whistle) the children should stop in place and remain until the evaluator notes the distance covered.

### Statistics Procedures

Initially, the Kolmogorov-Smirnov normality test was performed in all variables. All variables showed a normal distribution. For descriptive analysis, mean values, standard deviations, minimum and maximum were used in continuous variables and values of absolute and relative frequency in categorical variables. The variance difference between sexes was tested in all variables with the Student's T-test for independent samples. Associations were initially tested with Pearson's correlation test. Multicollinearity was previously tested and a high relationship was identified only between moderate-vigorous physical activity and cardiorespiratory fitness. These procedures were performed to meet the assumptions of multiple linear regression. The following analysis proposed to estimate the variance of standardized blood pressure from the other variables studied. Different multiple linear regression models were tested using the Stepwise method and those with the highest values of adjusted R^2^ were considered, as long as they maintained the theoretical logic. All associations were adjusted for sex, age and maturity-offset in order to remove the effect of the possible confounder. For all analyses, an alpha of 0.05 was considered a priori. The analyses were performed with the SPSS software for Windows version 20.0.

## Results

In the sample 53.5% are boys and 46.5% are girls. Table 1 shows the characteristics of the research subjects in relation to the studied variables. Only the variables: body fat percentage, moderate-vigorous physical activity and cardiorespiratory fitness showed differences between sexes (p<0.05). The low performance in the six-minute running/walking test in both sexes is highlighted.

**Table 1:** Description of research subjects characteristics (n = 215)

	Boys				Girls				
	n	X ± SD	Mín	Máx	n	X ± SD	Mín	Máx	p-value
**Age (years)**	115	8.25 ±1.54	6	12	100	8.51 ± 1.44	6	11	0.211
**SBP (mmHg)**	115	103.04 ± 11.35	83	143	100	103.81 ± 11.75	77	134	0.628
**DBP (mmHg)**	115	60.78 ± 9.13	41	81	100	60.67 ± 8.59	42	82	0.926
**Height (cm)**	111	134.27 ± 10.09	111	161	98	134.47 ± 10.84	108	154	0.890
**Weight (kg)**	111	32.98 ± 9.58	18	61	98	33.82 ± 11.23	15	67	0.558
**WC (cm)**	111	63.41 ± 9.09	48	86	98	61.75 ± 10	35	90	0.209
**BF%**	55	31.86 ± 9.09	15.6	51.2	57	35.24 ± 7.55	17.2	49.6	**0.035**
**BMI (kg/m²)**	111	18 ± 3.47	12.4	29.5	98	18.27 ± 3.98	12.6	29.78	0.600
**WHTR**	111	0.47 ± 0.05	0.37	0.64	98	0.45 ± 0.05	0.27	0.60	0.800
**MVPA (min)**	57	70.26 ± 29.57	23.62	147.16	60	55.33 ± 18.75	23.64	110.24	**0.002**
**CRF (m)**	101	800.43 ± 142.99	438	1158	91	749.25 ± 104.64	504	952	**0.005**

Source: the Authors. n: subjects number; X: average; SD: standard deviation; SBP: systolic blood pressure; DBP: diastolic blood pressure; WC: waist circumference; BF%: body fat percentage; BMI: body mass index; WHTR: waist-height ratio; MVPA: moderate-vigorous physical activity; CRF: cardiorespiratory fitness.

The correlation analysis is shown in table 2. Standardized blood pressure is associated in a negative and positive way with some variables in boys, however with reasonably low correlation (r) indexes. In girls, all values are below 0.3, indicating that in the bivariate analysis, associations are weak.

**Table 2:** Correlation values between standardized blood pressure and body composition, moderate-vigorous physical activity and cardiorespiratory fitness (n = 215)

	zBP	
	Boys (n:115)	Girls (n:100)
	r	r
WHTR	0.368	0.252
BMI	0.454	0.342
BF%	0.490	0.288
MVPA	-0.349	-0.253
CRF	-0.216	-0.227

zBP: standardized blood pressure; r: correlation coefficient; WHTR: Waist-height ratio; BMI: Body mass index; BF%: Body fat percentage; MVPA: moderate-vigorous physical activity; CRF: cardiorespiratory fitness.

After checking the correlations, adjusted association analyses were performed. In these analyses, the results demonstrate that considering sex, age and maturity-offset, the variables waist-height ratio, body mass index and body fat percentage indicated a positive association with standardized blood pressure, while moderate-vigorous physical activity and cardiorespiratory fitness indicated a negative relationship.

The predictor variables were tested in several models, however, three different models indicated the sets of factors that best explained the variance of standardized blood pressure (table 3). Although the three models are composed of different variables, they explained between 18 and 21% of the standardized blood pressure variance. Another highlight is that the two measures of nutritional status (body mass index and waist-height ratio) were the indicators that showed the highest magnitudes of association, considering the adjustments already described.

**Table 3:** Multiple linear regression to estimate the standardized blood pressure variability from 3 models (n = 215)

zBP			
	Model 1 (adjusted R^2^: 0.210)		
	β	p-value	CI 95%
WHTR	7.170	0.022	1.033 – 13.308
MVPA	-0.021	0.004	-0.035 – -0.007
Sex	-0.998	0.136	-2.315 – 0.320
Age	-0.302	0.212	-0.778 – 0.174
Maturity-offset	0.456	0.079	-0.054 – 0.965
	Model 2 (adjusted R^2^: 0.192)		
BMI	0,113	0,090	-0,018 – 0,245
MVPA	-0,023	0,002	-0,037 – -0,008
Sex	-0,697	0,408	-2,360 – 0,967
Age	-0,254	0,364	-0,808 – 0,299
Maturity-offset	0,260	0,471	-0,453 – 0,973
	Model 3 (adjusted R^2^: 0.183)		
BF%	0.054	0.043	0.002 – 0.107
CRF	-0.003	0.037	-0.006 – 0.001
Sex	-0.737	0.307	-2.163 – 0.689
Age	0.090	0.765	-0.508 – 0.688
Maturity-offset	0.238	0.466	-0.408 – 0.884

zBP: standardized blood pressure; adjusted R^2^: adjusted coefficient of determination to model variables; β: adjusted association for sex, age and maturity-offset; p-value: significance level; WHTR: Waist-height ratio; MVPA: moderate-vigorous physical activity; BMI: body mass index; BF%: body fat percentage; CRF: cardiorespiratory fitness.

## Discussion

The main results of the present study indicated that moderate-vigorous physical activity, cardiorespiratory fitness, anthropometric variables, age, sex and maturity-offset are important predictors of the standardized blood pressure variability in children. In our results, we observed that the systolic blood pressure mean was 103.0 mmHg in boys and 103.8 in girls and diastolic was 60.7 mmHg in boys and 60.6 in girls.

In the study by Gaya et al.^15^ with 416 Portuguese boys between 8-15 years old, the systolic blood pressure mean was 117.2 mmHg and diastolic blood pressure was 61.1 mmHg. In the study by Monteiro et al. (2015)^16^ with 51 Brazilian schoolchildren, the systolic blood pressure mean was 111.6 mmHg in boys and 107.6 in girls and diastolic was 66.8 mmHg in boys and 66.5 in girls aged between 13 and 16 years. A possible explanation for the systolic blood pressure values to be higher in the aforementioned studies compared to the findings may be due to the subjects’ chronological age being higher. This directly influences blood pressure levels, as older children tend to be higher, which naturally raises blood pressure levels.^2^

Regarding cardiorespiratory fitness, we noticed that the average number of meters covered in the six-minute test is low for boys and girls. In the study by Mello et al.^17^, although with children and adolescents between 10-17 years old, the prevalence of low cardiorespiratory fitness was 74.1% among young people, being higher in girls. Even using the PROESP-Br criteria, the same as in this study, the results found by Mello et al.^17^ were more worrying. However, in the studies by Coledam et al.^18^ and Minatto et al.^19^ the authors observed results similar to the present study, showing that around 50% of children have low levels of cardiorespiratory fitness.

Regarding moderate-vigorous physical activity, in the present study, we observed an average of 70.2 min/week in boys and 55.3 min/week in girls. Matsudo et al.^20^ observed in Brazilian children aged 9-11 years an average of moderate-vigorous physical activity of 59.5 min/day, with children accumulating more moderate-vigorous physical activity on weekdays than on weekends. In addition, 55.9% of children did not reach the daily recommendation.

We noticed that girls tend to have lower values of both cardiorespiratory fitness and moderate-vigorous physical activity. This data can be explained by a set of factors related to the culture of incentive to vigorous physical activities practice, anthropometric, ethnic and physiological variables^21^. Treuth et al.^22^ pointed out in a study carried out with girls, that the most performed activity during the week and on most weekend days are sedentary activities (55.4% of the time), in this active time, they have practised mostly activities with low intensity (41.7% light activities) and a little time in moderate (2.2%) and vigorous activities (0.7%).

Regarding associations, our initial results demonstrated a correlation between standardized blood pressure and all indicators of overweight and obesity, as well as moderate-vigorous physical activity and cardiorespiratory fitness. All results showed correlations with moderate or low magnitude (r <0.4). This low magnitude can be explained mainly by the several factors that influence blood pressure, such as height, sex and age,^23^ which were not part of this initial analysis. Therefore, from these first analyses, the need for adjusted analyses became clear.

In this perspective, models that can explain part of the standardized blood pressure variance by adjusting associations for the maturity-offset, age and sex were analysed. Thus, we perceive more clearly how much the variables: waist-height ratio, body mass index, body fat percentage, moderate-vigorous physical activity and cardiorespiratory fitness can in fact influence the standardized blood pressure of schoolchildren, both in an individually (through the Beta values) and jointly (through the adjusted R^2^ values).

From this, the predictor variables were tested and we arrived at three models that best explain the variance of standardized blood pressure. The first two models (model 1: waist-height ratio, moderate-vigorous physical activity, sex, age and maturity-offset; and model 2: body mass index, moderate-vigorous physical activity, sex, age and maturity-offset) explain 21% and 19% of the variance, respectively. The variable waist-height ratio, which is an indicator of abdominal fat, is the one that most influences the variability of blood pressure. These results are in line with the study by Silva et al.^24^ that showed an association of excess visceral fat with blood pressure levels and prevalence of arterial hypertension. However, according to the study by Cauduro et al.,^25^ overweight and obese children and adolescents were less likely to have high blood pressure levels regardless of sex, age and socioeconomic status, if they complied with the recommendations for physical activity for their age.

Therefore, the results of Cauduro et al.^25^ demonstrate the importance of considering physical activity and some indicator of overweight/obesity in the same association model in the trying perspective for understanding the set of predictors of blood pressure in children. Our results show that, regardless of the overweight/obesity indicator inserted in the model, the associations remain. This is justified by the fact that both variables are independently associated with blood pressure.^6^^,^^26^^,^^27^

In the last model presented, we observed that cardiorespiratory fitness and body fat percentage explain 18% of the standardized blood pressure variance. It is important to point out, although apparently, the magnitude of the association of cardiorespiratory fitness is low in relation to standardized blood pressure (β: −0.003), we realize that by the unit of measurement of this variable (meters) it is an important result and can easily influence at children's blood pressure.

Finally, it is important to add that cardiorespiratory fitness and moderate-vigorous physical activity are not in the same model because theoretically they are associated^28^, just as, when tested for multicollinearity, the relationship found in the present study was r>0.7. It is also important to note that even with three indicators of overweight/obesity performed using different measures (body growth, abdominal fat and image examination) the associations remained and the models maintained a similar percentage of standardized blood pressure explanation. In addition, we also emphasize that in the school environment the use of anthropometric measures to estimate children's nutritional status is an effective strategy and that it has an analysis effect similar to the image examination. This indicates that these variables (body mass index and waist-height ratio) can be added to intervention programs.

## Conclusion

We noticed that the body variables: body fat percentage, body mass index and waist-height ratio were shown to be influential to blood pressure. In addition, it was observed that moderate-vigorous physical activity and cardiorespiratory fitness, which are important variables related to exercise, were also shown to influence blood pressure in children. Therefore, it is concluded that all the studied indicators, when analysed together, are associated with the children blood pressure, suggesting that the early prevention of arterial hypertension in children consider the regular practice of moderate-vigorous physical activity, the increases in cardiorespiratory fitness levels and strategies for controlling of overweight and obesity indicators.
